# Case Report: Full-thickness prolapse of a neovagina created from sigmoid colon 25 years post-vaginoplasty for Mayer-Rokitansky-Küster-Hauser

**DOI:** 10.3389/fmed.2025.1573324

**Published:** 2025-12-01

**Authors:** Lijun Chen, Lin Sun

**Affiliations:** 1 College of Clinical Medicine, Jining Medical University, Jining, Shandong, China; 2 Affiliated Hospital of Jining Medical University, Jining, Shandong, China

**Keywords:** MRKH syndrome, sigmoid vaginoplasty, neovaginal prolapse (NP), full-thickness prolapse, long-term complications

## Abstract

Vaginoplasty is an effective treatment for patients with congenital vaginal absence, such as those with Mayer-Rokitansky-Küster-Hauser (MRKH) syndrome. Sigmoid vaginoplasty, a widely used surgical technique for vaginal reconstruction, is associated with satisfactory long-term outcomes. This paper presents a case of a patient who underwent vaginoplasty 26 years after the initial procedure due to complications arising from neovaginal prolapse of the sigmoid colon. The aim is to provide insights that may guide the management of patients following vaginoplasty.

## 1 Introduction

Mayer-Rokitansky-Küster-Hauser (MRKH) syndrome is a rare congenital developmental anomaly affecting females, with a prevalence ranging from 1 in 5,000 to 1 in 4,000. It is often associated with the congenital absence of the uterus or the presence of a rudimentary uterus due to bilateral hypoplasia of the paramesonephric ducts, affecting the uterus and the upper two-thirds of the vagina. In recent years, sigmoid colon vaginoplasty has been recognized as a viable option for treating vaginal agenesis due to its ability to maintain normal vaginal morphology and function over the long term (1, 2). The primary complications reported include issues related to poor bowel anastomosis and postoperative vaginal odor. However, there is evidence in the literature suggesting that vaginal prolapse may also be a potential complication of sigmoid colon vaginoplasty (3–7). Neovaginal prolapse of the sigmoid colon is relatively rare, with limited reports on its management (4, 8).

Neovaginal prolapse can be categorized into three types: mucosal prolapse, full-thickness prolapse, and vaginal vault prolapse. Mucosal prolapse is significantly more common and can typically be managed through surgical excision and electrocautery (3, 4). However, due to the rarity of full-thickness prolapse, no standardized treatment guidelines exist. Therefore, we present a case of severe full-thickness prolapse occurring 25 years after sigmoid vaginoplasty to contribute to the understanding of neovaginal prolapse management.

## 2 Case report

**Figure 1:**
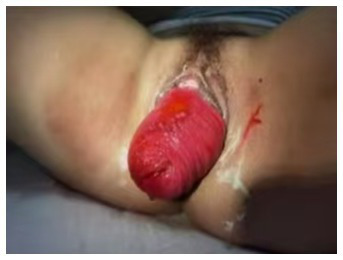
Neovaginal prolapse protruding approximately 5 cm beyond the hymen.

The patient, a 46-year-old female, was admitted to the hospital on July 21, 2024, with a chief complaint of a “prolapsed vulvar mass persisting for eight years.” She had previously undergone abdominal neovaginal reconstruction using a pedicled sigmoid colon graft 25 years earlier due to Mayer-Rokitansky-Küster-Hauser (MRKH) syndrome. Following the surgery, she married at the age of 24 and reported normal sexual intercourse. Eight years ago, she first noticed a vaginal mass while bathing, without any apparent triggering factor. The mass measured approximately 2 cm × 1.5 cm and was accompanied by a sensation of lower abdominal distension and excessive vaginal discharge. However, she did not seek medical evaluation or treatment at the time. Over the past 2 years, she experienced an increase in the size of the mass to approximately 5 cm × 3 cm, along with sexual discomfort. On physical examination, her secondary sexual characteristics were within normal parameters. A bright red protuberance, measuring approximately 5 cm × 3 cm, was observed at the external vaginal orifice. The surface of the mass was intact, with no signs of ulceration, though mild tenderness was noted. Clinical examination in the dorsal lithotomy position revealed eversion of the neovaginal mucosa, with the prolapse extending approximately 5 cm beyond the hymenal ring during the Valsalva maneuver (Figure 1). The uterus was not palpable on bimanual examination, and no abnormalities were detected in the adnexa. The Pelvic Organ Prolapse Quantification (POP-Q) system assessment yielded the following scores: Aa +3, Ba +5, C −15, GH 6, PB 3, TVL 20, Ap +3, Bp +5.

**Figure 2:**
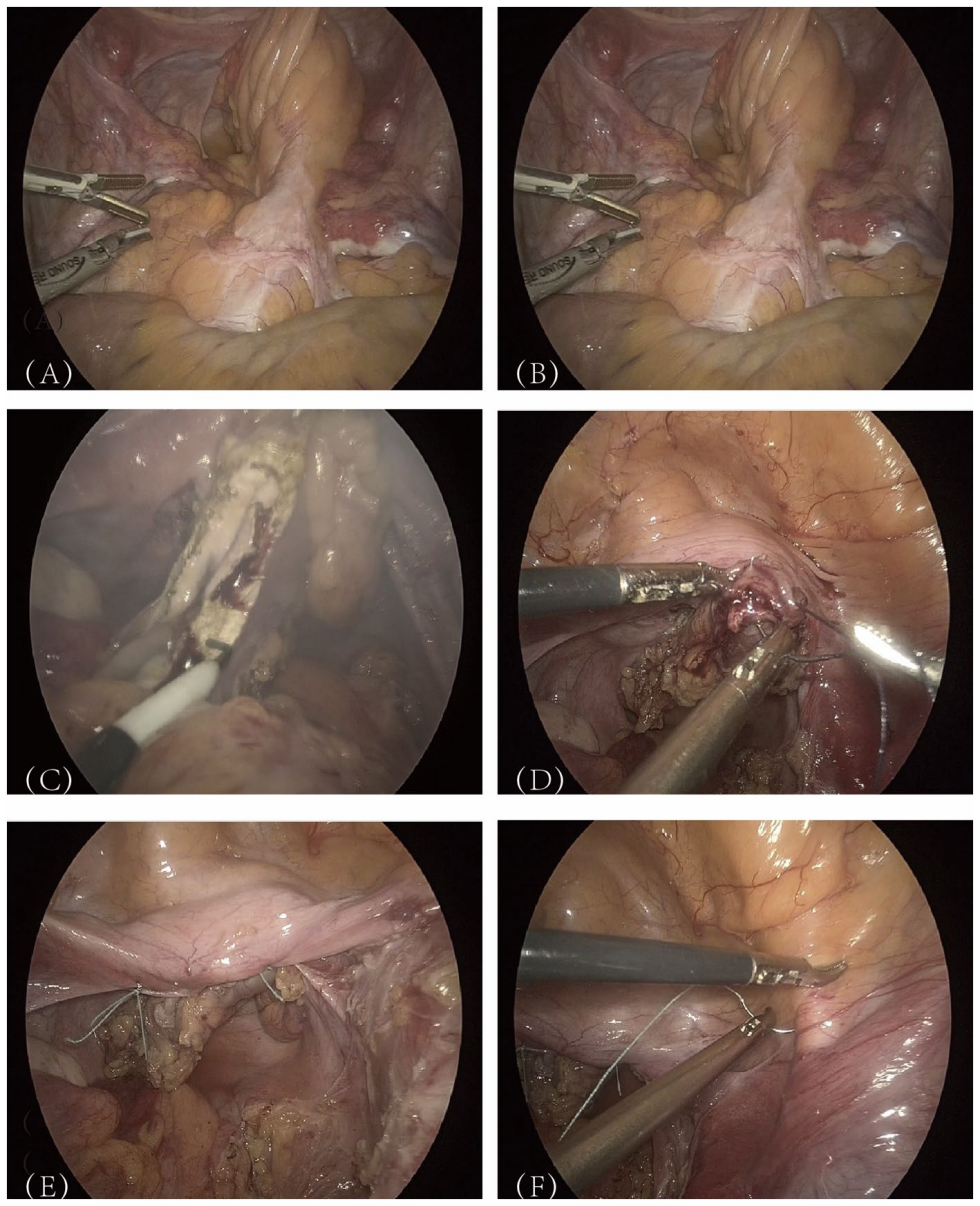
Presentation of key surgical steps.

Following the completion of preoperative evaluations, the patient was informed of the potential risks associated with the surgical intervention and provided with an informed consent form. After ruling out surgical contraindications, the procedure—“laparoscopic vaginal suspension + laparoscopic partial vaginal resection + laparoscopic pelvic adhesion release”—was performed on July 24, 2024. The patient was placed in the lithotomy position, and following the induction of general anesthesia, the surgical field was routinely sterilized, and sterile drapes were applied. Intraoperative exploration of the pelvic and abdominal cavities revealed extensive “cobweb-like” adhesions, with the greater omentum extensively adhered to the upper abdominal wall peritoneum, covering the pelvic floor. The peritoneum and intestinal structures on both sides of the pelvic cavity were adherent, forming a dense membranous encapsulation around the bilateral fallopian tubes and ovaries. No space-occupying lesions were identified. First, perform adhesiolysis in the pelvic and abdominal cavities to restore normal anatomy. A meticulous examination of the neovagina revealed that the sigmoid colon, approximately 22 cm in length, had been used as a vaginal substitute and exhibited dense adhesions with the surrounding tissues (Figure 2A). Further adhesiolysis was performed with meticulous identification and preservation of the ureters. The neovaginal bowel segment was mobilized and its blind end was exposed (Figure 2B). The mesentery surrounding the neovaginal bowel was progressively divided toward the pelvic floor. After isolating and transecting the relevant mesenteric blood vessels, approximately 8 cm of the neovaginal bowel segment was preserved, and the remainder was resected (Figure 2C). The inter-uterine strip structure was firm and provided adequate support, making autologous tissue suspension feasible without the need for synthetic mesh. During the procedure, the patient’s family was consulted, and a decision was made not to use mesh reinforcement. The severed end of the neovaginal bowel segment was closed with 1-0 absorbable suture (Figure 2D). It was then sutured to the bilateral rudimentary uterine horns using 1-0 non-absorbable braided polyester suture (Figure 2E). Following reinforcement, the neovagina was further suspended to the iliopubic ligaments (Figure 2F). The final measured vaginal depth was approximately 10 cm.

**Figure 3:**
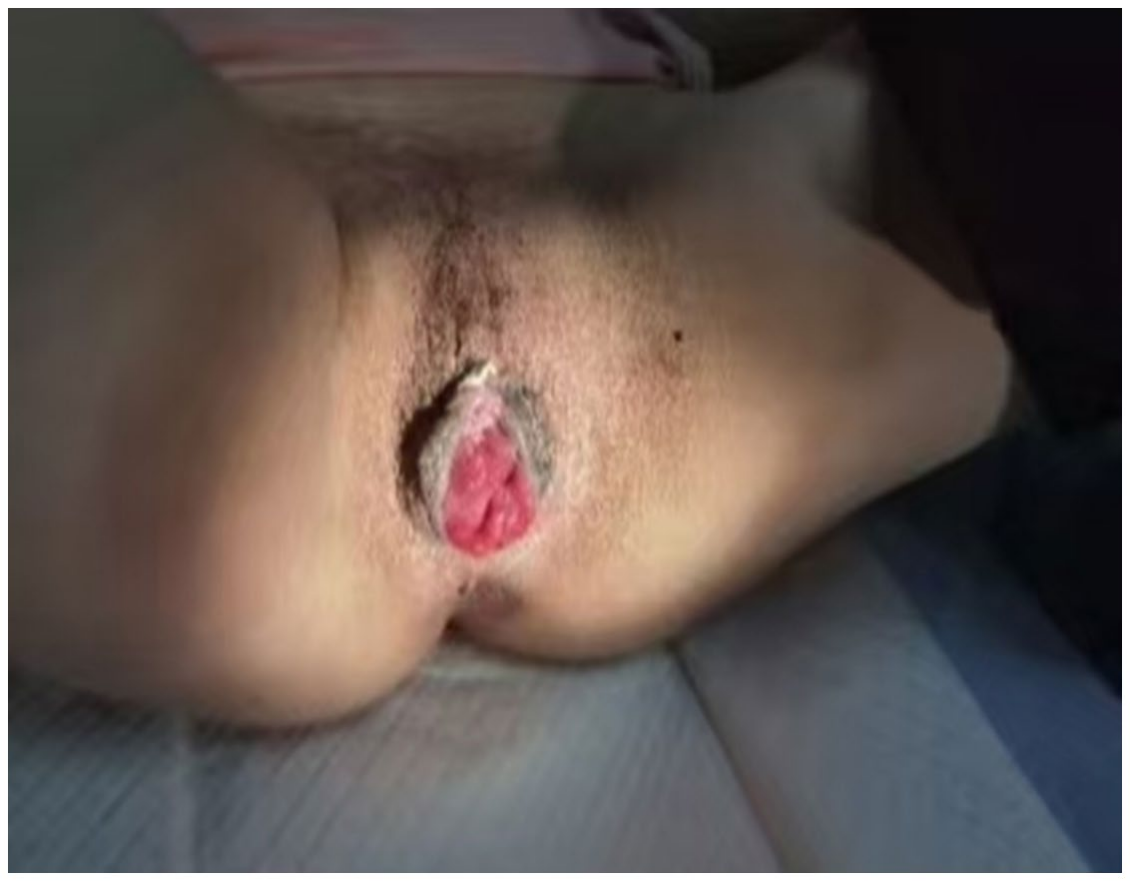
Patient’s recovery status 1 month post-surgery.

The operation was uneventful, with intraoperative blood loss estimated at 30 mL. A tissue sample from the intestinal wall was submitted for histopathological examination, which revealed interstitial vasodilatation and congestion. Postoperative anti-inflammatory therapy was administered, and the patient demonstrated a favorable recovery trajectory. A follow-up examination 1 month postoperatively showed that the vaginal opening had healed completely, The Pelvic Organ Prolapse Quantification (POP-Q) system assessment yielded the following scores: Aa −1, Ba −7, C −7, GH 6, PB 3, TVL 7, Ap −1, Bp −7, the vaginal mucosa remained intact, and vaginal secretions had improved significantly (Figure 3). The patient reported high satisfaction with her sexual life and did not experience any new discomfort. The one-year postoperative Pelvic Organ Prolapse Quantification (POP-Q) examination revealed findings consistent with the one-month assessment, and no new symptoms were reported.

## 3 Discussion

In patients with congenital absence of the vagina, the external genitalia are typically normal, and diagnosis is based on the presence or absence of the hymen, a shallow depression at the vaginal opening, or a short, shallow lower vaginal segment. According to the American College of Obstetricians and Gynecologists (ACOG), the initial approach to vaginal lengthening involves dilation (9). For patients in whom vaginal dilation is unsuccessful, several surgical techniques are available for neovaginal creation, including sigmoid vaginoplasty. There are relatively few reports of vaginal prolapse as a long-term complication of vaginal reconstruction. In one review, 179 patients who underwent sigmoid vaginoplasty as a vaginal replacement were followed, and nine cases developed vaginal prolapse of varying severity postoperatively (10). The etiology of neovaginal prolapse remains unclear. However, several possible mechanisms may contribute to its occurrence:

1. The neovagina is constructed from non-native tissue that lacks the intrinsic structural support of normal vaginal tissue and surrounding organs (11).
2. Suture-related factors may play a role, as the sigmoid colon used in the neovagina is typically sutured to the peritoneum. Over time, the peritoneum’s load-bearing capacity diminishes with aging, potentially leading to prolapse.
3. In some patients, an excessively long segment of the sigmoid colon is used for vaginal reconstruction. During postoperative sexual activity, fibrotic adhesions within the neovagina may cause the elongated segment to gradually descend under external mechanical stimulation.

Based on previous literature, vaginal vault or full-thickness prolapse is typically associated with a short neovaginal length created through mechanical dilation (12–15). A rare case of vaginal vault prolapse was reported following sigmoid vaginoplasty in a patient with a neovagina measuring 5–6 cm in length (16). The onset of neovaginal prolapse has been documented from the immediate postoperative period to as late as 33 years postoperatively (5). In our case, which occurred 25 years postoperatively, the development of neovaginal prolapse may have been associated with the progressive loosening or stretching of paravaginal support tissues over time.

The primary goals of treatment for neovaginal prolapse are the permanent correction of prolapse, maintenance of the proper anatomical position of the vagina, and normalization of sexual function (8). Due to the low prevalence of this condition, no standardized surgical approach has been established. Current evidence suggests that sacrospinous ligament fixation and sacrococcygeal vaginal fixation yield favorable outcomes in such cases (17). Yadav et al. (18) reported a case of vaginal prolapse occurring 22 years after sigmoid colon substitution of the vagina, in which laparoscopic sacrococcygeal vaginal fixation was performed with successful results. The sacrospinous ligament is known to be the strongest supportive ligament in the pelvis, and sacrospinous ligament suspension has demonstrated a high long-term success rate (>90%) for the correction of neovaginal prolapse (19). Postoperative outcomes are generally favorable, with minimal complications and a low recurrence rate. Several studies have highlighted the benefits of transcervical sacrospinous ligament fixation for prolapse correction following both non-surgical and surgical vaginoplasty, particularly in cases of MRKH syndrome with pelvic kidneys or failed prior abdominal surgery. However, a review of the literature suggests that transvaginal sacrospinous mesh repair may further reduce the incidence of recurrent vault prolapse and dyspareunia (19). In this patient, an abdominal approach was selected. The iliopubic ligament was chosen as the fixation point due to the presence of dense adhesions in the presacral area. Follow-up results were favorable, and the patient reported high satisfaction, indicating that this technique may serve as a reference surgical method for the treatment of vaginal prolapse.

One reason for not using a mesh intraoperatively was that the primitive basal inter-uterine strips were stiffer and provided sufficient support, allowing for suspension using the patient’s own tissues. Another drawback of mesh placement is that sutures can cause vaginal discomfort and interfere with sexual intercourse. Additionally, vascularization of the neovagina originates from the posterior vaginal wall. Therefore, placing a mesh may disrupt blood supply to the sigmoid neovagina (5). Patch repair is contraindicated in patients who have undergone enterovaginoplasty for neovaginal creation due to the increased risk of mesh exposure and infection (20).

A well-considered selection of vaginoplasty technique, proper intraoperative fixation of the neovagina, and appropriate postoperative care can help prevent neovaginal prolapse. Firstly, multiple surgical techniques are available for vaginoplasty in MRKH syndrome, including amniotic grafting, skin flap grafting, colonic substitution, and peritoneal vaginoplasty (3–6). Each method has its own advantages and limitations. However, the ideal vaginoplasty should prioritize safety, simplicity, and minimal disruption to vulvar anatomy. It should result in no visible scarring in the abdominal region, minimal post-molding vaginal atrophy, and reduced dependence on lubricants during sexual activity. It is reasonable to speculate whether this patient could have avoided or minimized the severity of prolapse if an alternative vaginoplasty technique had been chosen. The advantages of laparoscopic vaginoplasty have become increasingly apparent. Compared to open surgery, it offers minimal invasion, reduced risk of pelvic adhesions, shorter operative time, less bleeding, and faster recovery, making it a potentially preferred future option. In China, common techniques include bowel and peritoneal vaginoplasty. The latter provides superior cosmetic outcomes and higher sexual satisfaction due to the peritoneal properties of smoothness, moisture, and strong regeneration. However, as it requires longer vaginal stent use, the choice of procedure should be individualized based on patient needs, surgical expertise, and specific clinical conditions.

Additionally, in sigmoid colon vaginoplasty, it is now hypothesized that the neovagina should be routinely fixed to the sacral promontory or posterior peritoneum intraoperatively, and that the bowel segment used for vaginal reconstruction should be maintained in a state of retrograde peristalsis whenever possible. Postoperatively, it is recommended that patients remain on bed rest for at least 1 week. Regular use of a vaginal stent or paired molds may facilitate neovaginal attachment to the pelvic sidewalls, thereby reducing or preventing the occurrence of vaginal prolapse.

## 4 Conclusion

This case report illustrates the diagnostic and surgical approach to neovaginal prolapse, highlighting the importance of intraoperative, case-specific modifications to the surgical protocol. The effectiveness of the intervention may be further improved by extending the follow-up period and optimizing postoperative management. Although neovaginal prolapse is a rare condition, its impact on patients’ physical and mental well-being should not be underestimated. The accumulation of case reports and further research will aid in the development of standardized treatment and prevention strategies, ultimately providing patients with more effective therapeutic options.

## Data Availability

The original contributions presented in the study are included in the article/supplementary material, further inquiries can be directed to the corresponding author.
